# Gene dosage adaptations to mtDNA depletion and mitochondrial protein stress in budding yeast

**DOI:** 10.1093/g3journal/jkad272

**Published:** 2023-12-21

**Authors:** Joshua T McNamara, Jin Zhu, Yuhao Wang, Rong Li

**Affiliations:** Center for Cell Dynamics and Department of Cell Biology, Johns Hopkins University School of Medicine, Baltimore, MD 21205, USA; Biochemistry, Cellular and Molecular Biology (BCMB) Graduate Program, Johns Hopkins University School of Medicine, Baltimore, MD 21287, USA; Mechanobiology Institute, National University of Singapore, Singapore 117411, Singapore; Center for Cell Dynamics and Department of Cell Biology, Johns Hopkins University School of Medicine, Baltimore, MD 21205, USA; Biochemistry, Cellular and Molecular Biology (BCMB) Graduate Program, Johns Hopkins University School of Medicine, Baltimore, MD 21287, USA; Center for Cell Dynamics and Department of Cell Biology, Johns Hopkins University School of Medicine, Baltimore, MD 21205, USA; Mechanobiology Institute, National University of Singapore, Singapore 117411, Singapore; Department of Biological Sciences, National University of Singapore, Singapore 117411, Singapore

**Keywords:** mtDNA, proteostasis, mitochondria, protein aggregates, stress resistance, functional genomics, copy number variation, respiration

## Abstract

Mitochondria contain a local genome (mtDNA) comprising a small number of genes necessary for respiration, mitochondrial transcription and translation, and other vital functions. Various stressors can destabilize mtDNA leading to mtDNA loss. While some cells can survive mtDNA loss, they exhibit various deficiencies. Here, we investigated the impact of proteotoxicity on mitochondrial function by inducing mitochondrial unfolded protein stress in budding yeast. This led to rapid mtDNA loss, but aerobic conditioning imparted transient resistance to mitochondrial protein stress. We present a quantitative model of mtDNA loss in a growing cell population and measure its parameters. To identify genetic adaptations to mtDNA depletion, we performed a genome-wide screen for gene dosage increases that affect the growth of cells lacking mtDNA. The screen revealed a set of dosage suppressors that alleviate the growth impairment in mtDNA-deficient cells. Additionally, we show that these suppressors of mtDNA stress both bolster cell proliferation and prevent mtDNA loss during mitochondrial protein stress.

## Introduction

Mitochondria are present in nearly all known eukaryotic cells. In most species the majority of mitochondrial proteins are encoded by nuclear genes, yet a subset of genes required for mitochondrial gene expression and respiration are encoded on a separate, multi-copy genome located within the organelle. mtDNA is organized in punctate nucleoprotein assemblies called nucleoids, which generally contain 1–20 copies of the genome along with polymerases, a histone-like protein, and other factors required for replication and gene expression (Gustafsson *et al*. 2016; Miyakawa 2017). While experiments in the budding yeast *Saccharomyces cerevisiae* showed that cells that fail to inherit mitochondria are inviable (McConnell *et al*. 1990), they are known to survive the loss of mtDNA and, consequently, respiratory function. Among others, *Chlamydomonas*, several types of yeast, and some mammalian cells can proliferate after losing all mtDNA (García *et al*. 2000; Bonnefoy and Remacle 2023). Cells with intact mtDNA are referred to as *ρ^+^* (rho^+^) cells, and those that have lost mtDNA are called *ρ*^0^ (rho^0^) cells (Chen and Clark-Walker 1999).

Depletion of mtDNA, either by inactivating mutations, chromosome loss, or limited replication, is associated with states of cellular stress and disease. For example, mtDNA depletion in peripheral blood was found to correlate with age and has independent associations with cardiovascular disease, frailty, cognitive decline, and all-cause mortality, after controlling for age and other standard risk factors (Mengel-From *et al*. 2014; Ashar *et al*. 2015, 2017). Mitochondrial chromosome aberrations are also associated with neurodegenerative diseases that feature protein aggregation. Temporal lobe samples from Alzheimer's patients show more mtDNA nicking, fragmentation, deletions, and lower copy number than age-matched controls (de la Monte *et al*. 2000), and brain regions exhibiting tau and TDP-43 pathology have lower mtDNA copy number than histologically normal sections from surrounding tissue or corresponding samples from healthy controls (Klein *et al*. 2021). Likewise, Parkinson's disease patients develop mtDNA mutations and depletion in both the substantia nigra and in peripheral blood more frequently than peers (Bender *et al*. 2006; Kraytsberg *et al*. 2006; Dölle *et al*. 2016; Pyle *et al*. 2016).

Perturbing mtDNA homeostasis in model organisms and in cultured cells recapitulates disease phenotypes, demonstrating that mtDNA depletion is not only a passive indicator of pathologies. The “mtDNA mutator” mouse, in which the mitochondrial DNA polymerase (PolgA) has been replaced by a lower fidelity variant, rapidly accumulates mtDNA mutations (Trifunovic *et al*. 2004). These mice have a substantially shorter lifespan and prematurely develop senescence phenotypes such as hair graying and loss, osteoporosis, reduced fertility, muscle atrophy, and heart enlargement (Kujoth *et al*. 2005). In cultured pancreatic beta cells, targeted mtDNA depletion impaired glucose-stimulated insulin secretion, resembling a common senescence phenotype observed in vivo (Nile *et al*. 2014). Depletion of mtDNA in neuronal SH-SY5Y cells induced tau oligomers (Weidling *et al*. 2020), a toxic species in Alzheimer's disease, and increased ApoE transcription by over 65-fold (Gabrielli *et al*. 2023).

Considering that mtDNA depletion induces disease phenotypes, genetic and pharmacological interventions that enhance mitochondrial homeostasis may be useful in preventing or treating diseases. Such interventions might increase mtDNA replication, prevent mtDNA loss, or impart adaptation to the mtDNA-depleted state. Several strategies to augment mtDNA copy number have been reported, and some with apparent therapeutic benefits. Overexpression of the mtDNA nucleoid protein Abf2 (Zelenaya-Troitskaya *et al*. 1998), the ribonucleotide reductase Rnr1 (Lebedeva and Shadel 2007), or DNA recombinase Mhr1 (Ling *et al*. 2019) boosts mtDNA copy number in yeast. TFAM, the mammalian homolog of the nucleoid protein, boosted mtDNA copy number when overexpressed in mice and alleviated the phenotypic burden of mtDNA point mutations (Filograna *et al*. 2019). Mice overexpressing human TFAM showed increased survival following myocardial infarction and a reduction in myocyte apoptosis and interstitial fibrosis compared to wild-type animals. Other studies have been conducted to improve the fitness of mtDNA-depleted cells, and several deletions, mutations, and gene dosage alterations are known to partially rescue the growth of *ρ*^0^ yeast (Dunn and Jensen 2003; Garipler *et al*. 2014; Akdoğan *et al*. 2016; Vowinckel *et al*. 2021).

We previously reported that many cytosolic misfolded proteins are imported into mitochondria for degradation by mitochondrial proteases (Ruan *et al*. 2017). Whereas the imported misfolding-prone proteins are cleared efficiently after an acute stress such as heat shock, prolonged proteotoxic stress leads to mitochondrial damage. Because heat shock and disruption of cytosolic chaperones can have a wide range of effects on cellular physiology, in a later study we designed a strategy to examine the specific consequences of continuous, high-level expression of a misfolding-prone protein in the mitochondrial matrix and found that this leads to protein aggregation within mitochondria, imparts a growth defect, and induces mtDNA loss (Ruan *et al*. 2020). In this study, we investigated how the accumulation of unfolded proteins in mitochondria destabilizes mtDNA, modeled the fitness consequences of mtDNA loss, and screened for genetic suppressors of mtDNA stress. We found that mitochondrial unfolded protein stress imparts a growth defect by increasing the fraction of slow-growing *ρ*^0^ cells in a population, that mtDNA-binding proteins colocalize with unfolded protein aggregates, and that several gene dosage increases can accelerate *ρ*^0^ cell growth. Interestingly, these gene dose increases impart resistance to mitochondrial protein stress, both by reducing mtDNA loss and increasing the growth rate of cells following loss of mtDNA.

## Materials and methods

### Yeast strains

Yeast strains used in this study are derived from the *S. cerevisiae*BY4741 genetic background, and strain details are listed in Supplementary Table 3. Plasmid transformation was carried out using the lithium acetate method (Gietz and Schiestl 2007) and fluorescent labeling of endogenous proteins was achieved by genomic PCR-tagging (Sheff and Thorn 2004) or by selection from the GFP yeast collection (Huh *et al*. 2003). Gene knockouts were generated by homologous recombination, replacing a complete ORF with a PCR amplicon of the KanMX cassette flanked by homology arms. Cells were grown in yeast extract with peptone and D-glucose (YPD medium) at 30°C for all experiments, unless noted otherwise. Strains carrying plasmids were maintained in media containing 100 mg/L G418 (Roche) for selection during all experiments. Plasmids used in this work are listed in Supplementary Table 4.

### Imaging

Live cell microscopy was performed on a Carl Zeiss 200 m inverted microscope equipped with a Yokogawa CSU-10 spinning disk confocal unit, 100 × objective, 488 and 561 nm excitation lasers, and a Hamamatsu C9100-13 EMCCD detector. Any image panel featuring DAPI staining used fixed cells and was collected on a Nikon TE300 epifluorescence microscope equipped with a Nikon digital sight DS-Qi1MC camera using a 100 × objective and illuminated by LED. DAPI stained cells were prepared by inoculating 1 mL of YPD medium with cells from a yeast colony and incubating overnight. Cells were then sedimented by centrifugation at 3,000 *g* and resuspended in 100 μL of phosphate-buffered saline containing 4% formaldehyde. Fixation proceeded at room temperature for 15 minutes, after which cells were sedimented, washed with 1 mL tris-buffered saline (TBS), and again resuspended in 100 μL of a TBS solution containing 1% Triton X-100 and 200 ng/mL DAPI and incubated at room temperature for 30 minutes. Cells were again washed in 1 mL TBS before spotting on glass slides and imaging.

### Growth rate measurements

Before measuring growth rates, yeast strains were streaked on the appropriate selective agar plates and incubated for 2 days. These were then used to inoculate 5 mL YPD liquid cultures incubated in a rotary mixer for 16 hours. One microliter of each culture was then diluted into 199 μL YPD medium in flat-bottom, untreated 96-well polystyrene assay plates, mixed by pipetting and incubated at 30°C without agitation in a Tecan Infinite M200 Pro, with absorbance readings at 600 nm taken at 20 minute intervals. One replicate was conducted for the growth rate screen in Fig. 4e, and all other growth measurements were made with at least 3 replicates. Replicates constitute cultures inoculated from the same agar plate and handled independently for subsequent steps. Growth curves were log-transformed, truncated to the linear range, and fit with a linear model using an R script in order to determine growth rates. Where indicated, aerobic conditioning for growth rate measurements consisted of 2 days of culture in liquid YPG medium before inoculating YPD liquid cultures as described above.

### Petite colony assignment

To define a colony size threshold for assigning petite status, colonies of various sizes were assayed for growth on non-fermentable medium. Both mitoFluc and mitoCherry strains were spread at 1.5e^−5^ OD/plate on YPD agar in 10 cm petri dishes, incubated for 80 hours, and imaged on a document scanner. Colonies were segmented and measured from the cropped images using OpenCFU (Geissmann 2013) with an inverted threshold set to 2 and no radius filter. Cells from an arbitrary subset of colonies were then picked from the original petri dishes and patched on non-fermentable yeast extract, peptone, 3% glycerol medium (YPG), incubated for 4 days, and inspected for growth. This respiratory phenotype was then plotted against the colony area from the original YPD plate images. To determine whether this non-respiratory phenotype was associated with mtDNA loss, 10 colonies each from the respiring and non-respiring groups were selected arbitrarily from the original plates, stained with DAPI, and imaged as described above. A threshold of <1.5 mm^2^ was chosen for petite assignment in subsequent experiments, such that both strains had <50% respiring false-positives beneath the cutoff and few false-negative non-respiring colonies above it.

### mtDNA elimination

Elimination of mtDNA was carried out before each experiment to keep passage low and avoid suppressor mutations (Vowinckel *et al*. 2021). One microliter of cells from an exponential phase culture was diluted into at least 200 μL YPD + 200 μg/mL EtBr from a 5% (w/v) stock in 50% ethanol incubated for 1 day, passaged in fresh EtBr medium for 1 day, sedimented, resuspended in fresh YPD medium, and incubated for 1 day. Elimination of mtDNA was confirmed by the absence of growth of at least 1e^5^ cells spotted on YPG medium and incubated for 4 days.

### Time-resolved mtDNA loss measurement

All strains were grown in YPG medium for 3 days to enrich for *ρ^+^* cells. Cultures were then inoculated to an OD of 0.05 in YPD (8 replicates each, mitoCherry and mitoFluc strains) and maintained below a density of 0.5 by dilution with fresh medium for the duration of the experiment. After a 3 hour recovery period, 1.5e^−5^ OD units of cells were withdrawn from each culture and plated on 10 cm petri dishes of YPD medium at 3 hour intervals for 9 hours. Plates were incubated for 80 hours after inoculation and then imaged and analyzed as described above.

### MoBY plasmid library preparation

The MoBY-ORF library devised by Boone and colleagues (Ho *et al*. 2009) was purchased from Dharmacon in the form of *Escherichia coli* strains in an arrayed format bearing the plasmids of interest. The library was replicated to 96-well microplates containing 100 μL of LB medium + 10 mg/L chloramphenicol + 50 mg/L kanamycin per well and these cultures were incubated for 18 hours at 37°C in a humid chamber. Each culture appeared to reach saturation. All cultures were then pooled, and plasmids were isolated with a Qiagen plasmid Maxi kit.

### Yeast transformation

Three micrograms of the pooled plasmid library was transformed into *S. cerevisiae* strain BY4741 using a lithium acetate method optimized for libraries (Gietz and Schiestl 2007), except that the heat shock was carried out for 90 minutes. Sixteen sterile bioassay dishes (Sigma-Aldrich) measuring 245 × 245×25 mm were each filled with 150 mL YPD + agar medium including 100 mg/L G418 for selection. Three days after performing the transformation, plates were found to contain ∼12,000 colonies each. Colonies were resuspended in YPD + G418 medium and pooled together to form the inoculum for the barcode sequencing screen.

### Barcode sequencing experiments

To identify dosage suppressors of mtDNA stress, 2 successive experiments were carried out. Each experiment was performed in 3 independent replicates for all steps between the initial library transformation and the Illumina sequencing run, which were performed as single procedures. All cell count estimations were done by measuring culture absorbance at 600 nm, using an extinction coefficient of 1 OD = 1e^7^ cells.

For each replicate, 1e^8^ yeast cells bearing the pooled MoBY-ORF plasmid library were used to inoculate 100 mL YPD medium + 100 mg/L G418 and cultured for 24 hours at 30°C with double-orbital shaking at 220 RPM in 1 L flask. 1e^8^ of these cells were then transferred to fresh medium containing 10 mg/L ethidium bromide added and incubated for 24 hours. Two more passages were conducted in this fashion, and the resultant population did not contain any detectable respiration-competent cells, assessed by plating 1e^6^ cells on YPG medium. An aliquot of 1e^8^ cells was withdrawn from this culture, sedimented, and frozen for later analysis. Another aliquot of 1e^8^ cells was used to inoculate 100 mL YPD + G418 medium without EtBtr and passaged twice more as during EtBr treatment. After the third day of growth, 1e^8^ cells were withdrawn and frozen for analysis.

### Sequencing

Plasmids were isolated from thawed cell aliquots for sequencing using a Zymoprep Yeast Plasmid Miniprep II kit. Primers were designed (Supplementary Table 5) to amplify the barcode region of the plasmids and add an in-line Illumina index sequence for each replicate (see Supplementary Table 1 for index key), a Read 1 sequencing read primer site, and flanking flow cell adapter sequences. Twenty nanograms of each plasmid was used as a template in a 100 µL PCR using Q5 polymerase (NEB) and standard reagents and procedures. Amplicons were purified by agarose gel electrophoresis and a spin column cleanup kit (Qiagen) and quantitated using the Qubit fluorometric dye-binding assay (Thermo Fisher). Amplicons were pooled at equal concentrations and quality-checked by capillary electrophoresis on an Agilent Fragment Analyzer and sequenced on an Illumina Hi-Seq lane using standard reagents and protocols (Genewiz).

### Sequencing data analysis

Raw data processing and barcode indexing was performed using a custom shell script followed by an R script (see code repository for details). Reads that did not exactly match an experiment index sequence and a gene barcode sequence were discarded. Barcode enrichment was assessed using a binomial exact test as implemented in the edgeR package (Robinson *et al*. 2010). Two screens were analyzed. The first compared barcode abundances in cultures with and without ethidium bromide treatment and consequent mtDNA elimination. The second test compared cell populations after a *ρ*^0^ outgrowth phase to those harvested just after mtDNA elimination. Genes were then assigned a rank metric, which consisted of the sign of relative enrichment (1 if the gene was enriched or −1 if the gene was depleted) multiplied by the negative base-10 logarithm of the *P* value yielded by the binomial exact test. Genes that were enriched between the 2 time points thus had a positive rank metric and those that were depleted had a more negative rank metric. We used the Benjamini–Hochberg (BH) procedure to control for the false discovery rate (FDR) associated with our *P* values when determining significance.

### Growth rate screen

The top 147 most adaptive and 45 least adaptive plasmids from the *ρ*^0^ growth barcode sequencing screen (based on ranking metric) were recovered, along with an empty control plasmid, from stocks and re-transformed into yeast in an arrayed format using a Frozen-EZ Yeast Transformation II Kit (Zymo Research). Elimination of mtDNA and growth rate determination were carried out as described previously.

### TMRM staining

Three replicates each of *ρ*^0^ and *ρ^+^* cells were prepared from yeast strains bearing MoBY plasmids and grown in 1 mL YPD + G418 cultures in a 96-well block of 2 mL well volume for 24 hours in in a double-orbital shaking incubator set to 220 rpm. Cells were sedimented by centrifugation at 1,100 *g* for 3 minutes and resuspended in 200 μL PBS + 5% (w/v) D-glucose + 50 nM TMRM, then incubated for 10 minutes at 30°C. The cells were again sedimented and resuspended in the same buffer, but without TMRM. This suspension was then diluted 40-fold with additional buffer and injected on an Attune flow cytometer using an autosampler running at 100 μL/minute and read in the RL2 channel (638 nm excitation laser, 720/730 nm emission filter). At least 1e^5^ detection events were collected for each replicate. Data analysis was conducted using FlowJo software, and gates were applied to select a Gaussian peak containing the majority of events in the forward scatter and side scatter channels. Plotted data represent the mean fluorescence intensity of events from a cytometry run of 1 biological replicate.

### Petite frequency analysis

Petite frequency determination was performed using a protocol adapted from Hess *et al*. (2009). Yeast strains were streaked on YPD + G418 agar plates and incubated for 2 days, then these cells were used to inoculate YPG + G418 liquid cultures and incubated for 2 days in a rotary mixer. Cells from the YPG cultures were plated on YPD + G418 plates to a density of approximately 150 colonies per plate and incubated for 48 hours. Eight colonies from each plate were then resuspended in water, diluted, and used to inoculate new plates at a target density of 150 colonies per plate. These plates were then imaged, and colonies were counted and petites thresholded as described above. Each plate founded from an independent colony was considered a biological replicate.

### Split-GFP imaging

Split-GFP strains were constructed similar to Ruan *et al*. (2017). GFP1-10 was fused with the mitochondrial matrix protein Grx5 under a *GPD* promoter and stably integrated into the yeast genome at the *TRP1* locus. Endogenous *HSP104* or *SCY1* was tagged by GFP11 at the C-terminus and mitochondria were marked by Tom70-mCherry tagged at the endogenous locus. For quantification, confocal sections were maximum intensity projected in the z-axis and mitochondria were segmented in the mCherry channel by Otsu's method using an ImageJ plugin. Total GFP signal within the mitochondrial boundary for each cell was then summed.

## Results

### Mitochondrial unfolded protein stress causes mtDNA loss

**Fig. 1.**
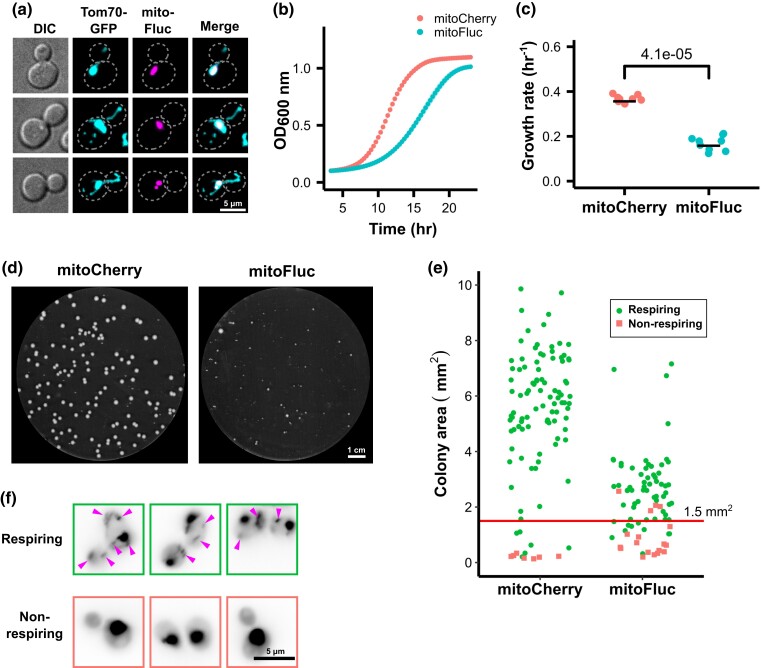
Mitochondrial unfolded protein stress impairs growth and induces mtDNA loss. a) Fluorescence image of yeast cells expressing an aggregation-prone, mitochondria-targeted synthetic protein (mitoFluc) tagged with mCherry and with the outer mitochondrial membrane protein Tom70 tagged with GFP. b) Growth curves of cells expressing either mitoCherry or mitoFluc. Optical density at 600 nm (OD600) was measured every 20 minutes; points represent means and shading (partly obscured) represents standard error of 8 biological replicates. c) Exponential growth rates of cells expressing either mitoCherry or mitoFluc. Constants determined by log-linear fit to the OD600 curves. Black bar indicates mean of 8 biological replicates. *P* value from 2-tailed *t*-test. d) YPD plate of yeast colonies expressing either mitoCherry or mitoFluc. e) Assay for respiratory competence by colony size. Each dot represents 1 yeast colony. After area measurement, colonies were patched on YPG plates to test for respiratory growth. Line at 1.5 mm^2^ indicates the threshold chosen to define the petite colony phenotype, which yields similar false-positive petite calls between the strains. Green circles represent respiring colonies, red squares represent non-respiring colonies. f) Representative images of cells from respiration-competent and respiration-negative colonies with DNA stained by DAPI. Magenta arrows indicate mtDNA nucleoids, and the large DAPI spots are cell nuclei.

We previously described the engineered protein mitoFluc, a fusion of an N-terminal mitochondrial targeting sequence with a misfolding-prone firefly luciferase variant (FlucSM) (Gupta *et al*. 2011) and a C-terminal mCherry tag (referred to as mitoFluc). This protein spontaneously forms aggregates (Fig. 1a) that also recruit other misfolded proteins within the mitochondrial matrix, which we termed deposits of unfolded mitochondrial proteins (DUMP) (Ruan *et al*. 2020). In this study, we employed the same system to disrupt mitochondrial proteostasis. When constitutively expressed in budding yeast, mitoFluc impairs proliferation, reducing the population exponential growth rate to approximately half that of control cells expressing mitoCherry, the same vector but lacking the part encoding FlucSM (Fig. 1, b and c). Unlike acute proteotoxic stresses such as a pulsed heat shock (Ruan *et al*. 2017), this system produces a chronic proteostasis burden. Induction of mitoFluc, which leads to the stable formation of protein aggregates in the matrix, increases the incidence of smaller colonies (Fig. 1d), which are known as “petites” and can be an outcome of mitochondrial DNA mutation or deletion.

To determine whether the petite colony phenotype was caused by loss of mtDNA, we measured the area of 186 colonies and then replated them on yeast medium containing glycerol as the major carbon source. Cells that contain nonfunctional mtDNA or have lost mtDNA completely are unable to respire and thus do not grow on this medium. As expected, petite colonies with an area < 1.5 mm^2^ were mostly unable to grow on the glycerol medium (Fig. 1e). To determine whether these colonies had completely lost mtDNA or had merely sustained a loss-of-function mutation in the respiratory chain, we selected 10 each of the respiring and non-respiring colonies and stained them with 4′,6-diamidino-2-phenylindole (DAPI). All of the respiration-competent cells contained cytoplasmic foci that stained with DAPI, indicative of mtDNA, yet none of the non-respiring colonies showed cytoplasmic staining, consistent with complete loss of mtDNA (Fig. 1f, Supplementary Fig. 1). We thus concluded that stress caused by unfolded proteins in the mitochondrial matrix induced mtDNA loss.

### Modeling and measurement of mtDNA loss during mitochondrial protein stress

**Fig. 2.**
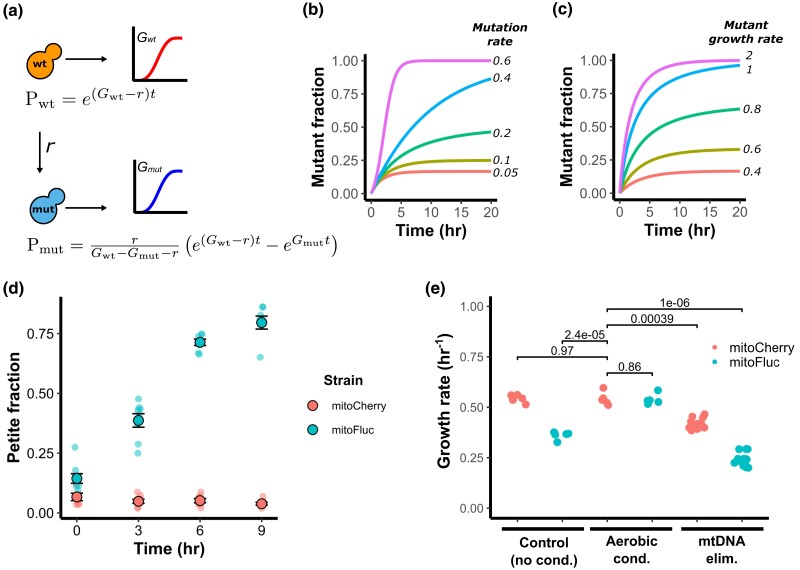
Modeling and determination of mtDNA loss rate in a growing cell population. a) Schematic of 2-state model of mtDNA loss in a growing population. *G_wt_* is the growth constant of the wild-type cells, *G_mut_* is the growth constant of the mutated *ρ*^0^ cells, *r* is the rate of mtDNA loss, *P_wt_* is the population size of wild-type cells, *P_mut_* is the population size of mutant *ρ*^0^ cells, *t* is time since the foundation of a population. b) Simulated population proportion of mtDNA mutants for a range of mutation rates and c) mutant growth rates. d) Time-resolved assay of mtDNA loss during mitochondrial protein stress. Cultures were kept in exponential growth phase for experiment duration by dilution with new media to avoid selecting for respiring cells. Circles represent measured petite frequencies in independent biological replicates, black circles indicate the population-weighted mean petite fraction from 8 biological replicates, error bars represent simple SEM of petite frequencies between each replicate (unweighted). e) Effect of mitochondrial modifiers on growth rate. Dots indicate rates from independent biological replicates. *P* values are calculated from a 2-tailed *t*-test.

Considering that *ρ*^0^ cells grow more slowly and produce smaller colonies, we wondered whether the protein stress had impaired population growth by increasing the fraction of *ρ*^0^ cells. We formalized this hypothesis as a simple 2-state growth model. In this model, a population of wild-type cells with a particular growth rate (*G_wt_*) loses mtDNA at a constant rate (*r*), giving rise to *ρ*^0^ mutant cells that have a different growth rate (*G_mut_*). The fraction of wild-type and *ρ*^0^ mutant cells in the population at a given time (*t*) is represented as *P_wt_* and *P_mut_*, respectively. Combining this system with the standard exponential growth equations yields the expressions in Fig. 2a (see Supplementary Fig. 2 for derivation). We can simulate several scenarios of interest by applying these equations. The first is that for a sufficiently high mtDNA loss rate, the population approaches complete *ρ*^0^ mutant dominance (Fig. 2b). Similarly, if the *ρ*^0^ mutant grows more quickly than the wild type, it will out-compete the wild-type population (Fig. 2c). However, if *G_wt_* − *r* > *G_mut_*, the population fraction of the mutant increases and then approaches equilibrium. This produces roughly fixed proportions of mutant and wild-type cells in the population and a constant population growth rate equal to the weighted average of the wild-type and mutant growth rates. This last scenario is consistent with the observation that mitoFluc expression reduces the overall population growth rate (Fig. 1b) and increases the proportion of *ρ*^0^ cells while still preserving a *ρ^+^* population after many passages (Fig. 1, d and e).

We performed experiments to test this model and obtain its parameters. To estimate the mtDNA loss rate, we subjected mitoFluc and mitoCherry cells to aerobic conditioning in glycerol medium (yeast extract with peptone and glycerol, YPG) to select a starting population of *ρ^+^* cells and then inoculated cultures in rich glucose medium (yeast extract with peptone and d-glucose, YPD) with these cells to relax selection for respiration. These cultures were maintained in the exponential growth phase by periodic dilution with fresh medium to prevent glucose depletion. Cultures were sampled at regular intervals and plated onto agar plates to allow isolated colonies to grow. We then measured the area of each colony on the plates and counted the number of petite colonies, defined as those with an area < 1.5 mm after 80 hours of growth, to obtain the petite fraction for each strain over time. Following aerobic conditioning, mitoFluc cultures had a petite fraction that was similar to mitoCherry control cells (Fig. 2d). After relaxation of respiratory selection, the control population did not accumulate petite colonies above the baseline level, yet the mitoFluc petite fraction increased in an approximately linear fashion, exceeding 75% after 9 hours.

If the growth defect caused by mitochondrial protein stress is entirely due to mtDNA loss, selection for respiring *ρ^+^* mitoFluc cells should raise the population's growth rate to that of the wild type. Subsequent passage on nonselective medium would allow the population to accumulate non-respiring *ρ*^0^ cells and its growth rate would diminish in proportion. Furthermore, complete elimination of mtDNA by chemical means should reduce the growth rate of both wild-type and mitoFluc cells to an equal rate that is slower than a mitoFluc population from a regular culture, which contains a mixture of *ρ*^0^ and *ρ^+^* cells. We measured the growth rates of mitoFluc and control populations after normal passage in glucose medium, after aerobic conditioning in glycerol medium, and after complete mtDNA elimination by treatment with ethidium bromide (EtBr). Nonconditioned mitoFluc cells exhibited a growth defect relative to a control. However, the growth rate of mitoFluc cells was boosted to the wild-type level after aerobic selection for *ρ^+^* cells (Fig. 2e). Chemical elimination of mtDNA from wild-type and mitoFluc cells substantially reduced their growth rates. Yet *ρ*^0^ wild-type cells grew faster than unconditioned mitoFluc cells, and *ρ*^0^ mitoFluc cells grew slower still. It appears that the overall population growth rate within each strain is consistent with impairment due to mtDNA loss, but that mitochondrial protein stress depresses *G_mut_*, the growth rate of *ρ*^0^ cells, beyond the growth defect caused by mtDNA loss alone. This indicates that mtDNA loss only partly accounts for the growth defect imparted by mitochondrial protein stress.

### Mitochondrial protein aggregates sequester mtDNA-binding proteins

**Fig. 3.**
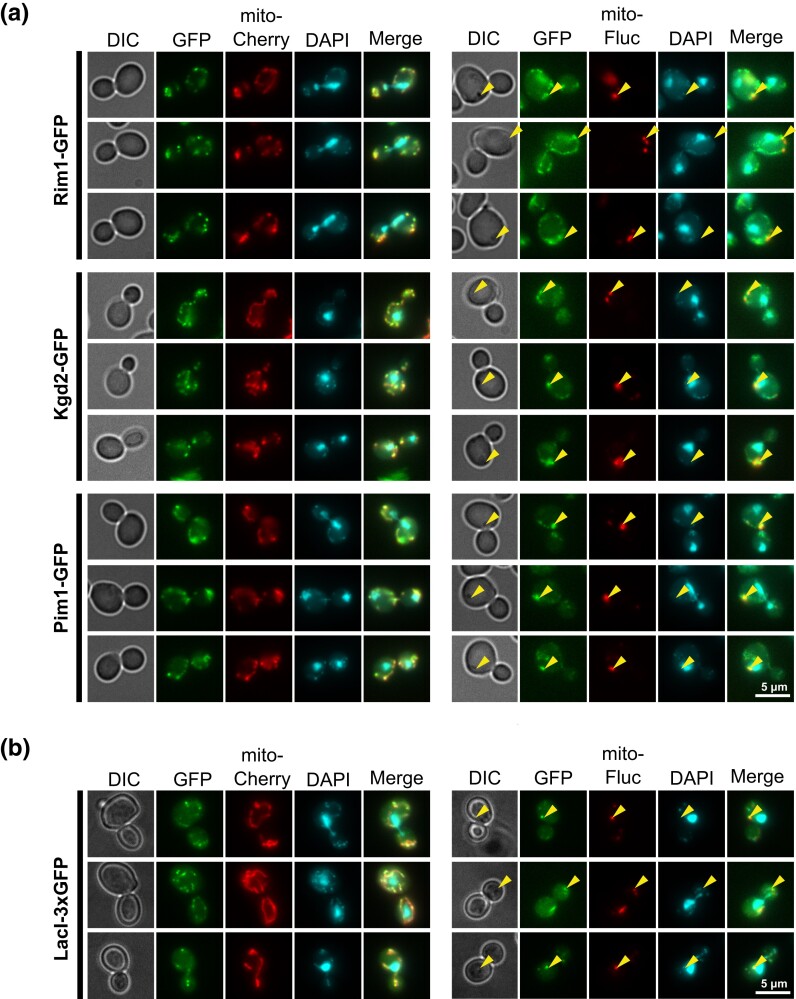
mtDNA-binding proteins are enriched in mitochondrial protein aggregates. a) Representative fluorescence images of either mitoCherry control cells or mitoFluc cells grown in YPD medium with various mtDNA-binding proteins tagged with GFP. b) Fluorescence micrograph of mtDNA-LacO LacI-3xGFP mitoCherry or mitoFluc cells stained with DAPI.

We next asked how mitochondrial protein stress could induce mtDNA loss. One possibility is that unfolded or aggregated proteins sequester proteins required for mtDNA maintenance. To test this, we tagged the known mtDNA-binding proteins Rim1, Kgd2, and Pim1 (Kaufman *et al*. 2000; Kunová *et al*. 2017) with GFP and imaged them in the presence and absence of mitoFluc protein aggregates. In wild-type cells, the GFP-tagged proteins colocalized with the mCherry signal that marked the mitochondrial matrix and were enriched at mtDNA nucleoids, as indicated by DAPI staining. In cells bearing mitoFluc aggregates, the GFP-tagged proteins can be found at mtDNA nucleoids, although in many instances they colocalized with mitoFluc aggregates where no DAPI signal was detected (Fig. 3a). A bright focus of mtDNA-binding proteins in each cell colocalized with a mitoFluc aggregate rather than any nucleoid that is not associated with mitoFluc aggregates.

To determine whether sequestration by mitoFluc aggregates is a general tendency of mtDNA-binding proteins, we introduced a LacO-LacI-GFP system into cells expressing mitoFluc. An array of LacO sequences was spliced into the mitochondrial genome and the LacI protein, which binds to LacO sequences, was fused to a mitochondrial targeting sequence and 3xGFP repeat and expressed from the nuclear genome (Osman *et al*. 2015). Interestingly, like the mtDNA-binding proteins, LacI-3xGFP colocalized with mtDNA, but in cells expressing mitoFluc a substantial fraction of LacI-3xGFP colocalized with mitoFluc aggregates in positions where no mtDNA was detected (Fig. 3b). Thus, this ectopic DNA-binding protein displaces from mtDNA and localizes to misfolded protein aggregates during mitochondrial unfolded protein stress, similar to native mtDNA-binding proteins.

### Whole-genome screen for dosage suppressors of mtDNA stress

**Fig. 4.**
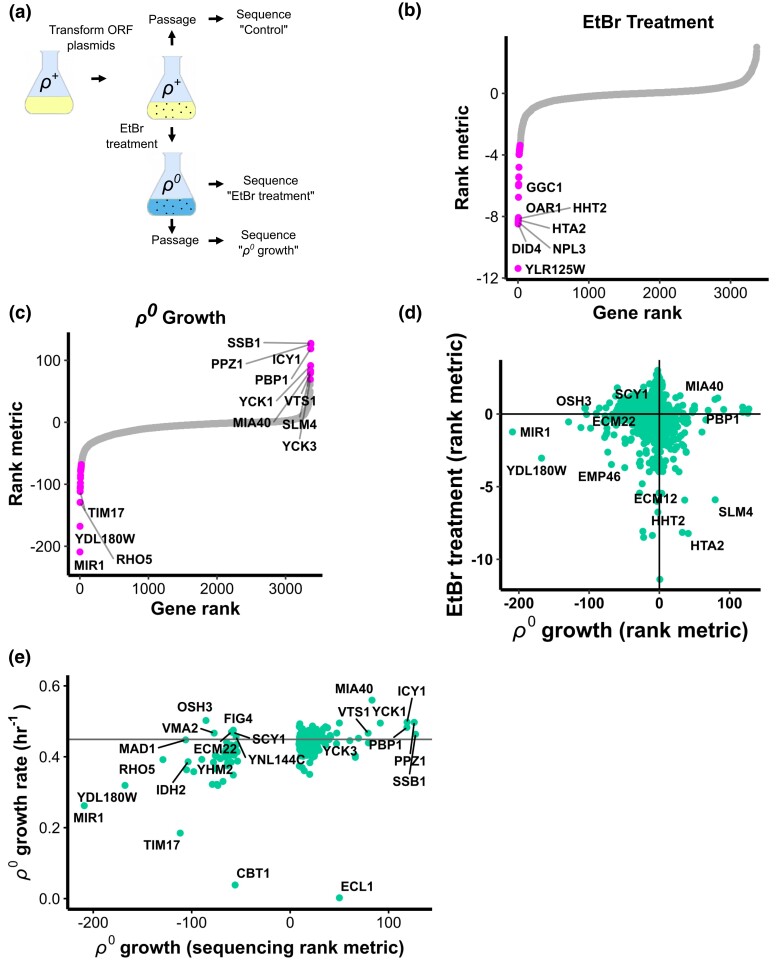
Genome-wide screen for dosage suppressors of mtDNA stress. a) Schematic of how 3 samples were prepared for barcode sequencing screen. b) Enrichment of gene dosage increase plasmids during EtBr treatment. Rank metric is the negative, base-10 logarithm of the raw *P* value associated with a gene's differential enrichment multiplied by the sign of the logarithm of its fold-change (i.e. negative for plasmid depleted and positive for enriched). Genes are rank ordered along the *x* axis. *P* values calculated from 3 biological replicates. Genes for which *P* < 0.05 after applying Benjamini–Hochberg (BH) correction for false discovery feature magenta dots. c) Enrichment of gene dosage increase plasmids during *ρ*^0^ growth. Plot elements as in B, except that points are magenta for *P* < 10^−65^ after BH correction. d) Comparison of EtBr and *ρ*^0^ growth experiments. e) Summary plot comparing *ρ*^0^ growth barcode sequencing screen and *ρ*^0^ growth rate screen results. Horizontal line indicates the growth rate of the control strain.

Cells that have lost mtDNA possess all of the biochemical faculties necessary to survive in glucose-rich medium, yet some process becomes rate-limiting and impairs growth. In order to identify pathways related to this deficit and identify genes for which elevated function may suppress defects caused by mtDNA loss, we conducted a genome-wide dosage-dependent suppressor screen for genes that accelerate the growth of *ρ*^0^ cells (Fig. 4a). We employed the Molecular Barcoded Yeast Open Reading Frame (MoBY-ORF) library to create gene dosage variation in a population of budding yeast cells (Ho *et al*. 2009). This is a collection of centromeric plasmids that contains 4,981 budding yeast ORFs, each flanked by its native promoter, terminator, and a unique oligonucleotide barcode. The library was pooled and transformed into yeast, and the resulting culture was split into 2 groups consisting of 3 replicates each. The first group of control cells was passaged in regular YPD growth medium and harvested, while the other group was treated with EtBr to eliminate mtDNA. An aliquot was drawn for analysis, and then this sample was passaged in YPD medium to allow proliferation of each clone according to its *ρ*^0^ fitness. These *ρ*^0^ cells were then harvested and plasmids were purified from all samples for sequencing.

Enrichment of MoBY gene plasmids was determined by comparing barcode frequencies from the control cell populations to barcode frequencies after EtBr treatment. Then, a second comparison was drawn between this EtBr-treated group and its *ρ*^0^ progeny that were passaged for a growth period. Using EtBr-treated cells as the basis for this comparison enables identification of adaptations to *ρ*^0^ growth without enriching for adaptations to the EtBr treatment itself. Differential barcode enrichments resulting from these treatments were calculated using a negative binomial model from the edgeR software package (Robinson *et al*. 2010). Plasmid barcodes that become more frequent after a treatment correspond to an adaptive gene dose increase, and those that become less frequent correspond to gene dose increases that are maladaptive relative to the rest of the population. Differentially enriched barcodes were identified for both EtBr treatment and *ρ*^0^ growth (Fig. 4, b and c), yet adaptations to each condition were distinct and bore few similarities (Fig. 4d). The largest effects in the EtBr treatment were produced by maladaptive plasmids, and no plasmids imparted a significant adaptation with *P* < 0.05 after correcting for false discovery. Both *HHT2* and *HTA2*, which encode histone core proteins H3 and H2A, potently repressed growth during EtBr treatment. Their genomic duplicates, *HHT1* and *HTA1* respectively, were not detected at baseline or after treatment and could not be analyzed (Supplementary Table 1).

Some of the genes that provided the most robust adaptation to *ρ*^0^ growth after EtBr treatment were previously reported in similar studies, including *MIA40*, *PPZ1*, *SSB1*, *ICY1*, and *PBP1* (Dunn and Jensen 2003; Garipler *et al*. 2014; Akdoğan *et al*. 2016; Vowinckel *et al*. 2021). We found several high-confidence adaptative genes not reported by existing literature, namely *VTS1*, *YCK1*, *YCK3*, and *SLM4* (Fig. 4c).

### Dosage suppressors of mtDNA stress alleviate mitochondrial protein stress and reduce mtDNA loss during protein stress

To evaluate the results of the barcode sequencing screen using another assay technique, we reconstructed dosage-increase strains for 147 adaptive and maladaptive genes using their source plasmids and measured their growth rates in an arrayed format both before and after mtDNA elimination by EtBr treatment. We then compared these growth rates to that of a control strain bearing a MoBY plasmid not carrying any yeast ORF. These measurements broadly replicated the results from the primary barcode sequencing screen (Fig. 4e), and *ρ*^0^ cell growth rates moderately correlated with the barcode sequencing rank metrics (Pearson's *r* = 0.42).

**Fig. 5.**
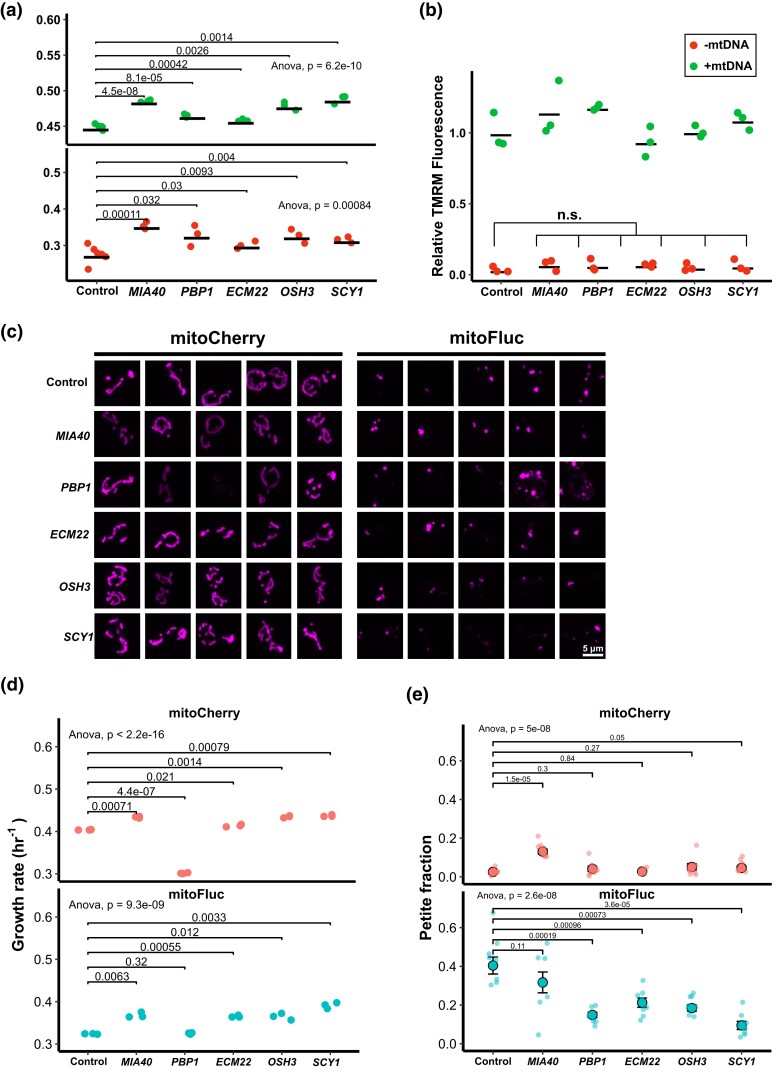
*ρ* ^0^ growth enhancers increase growth rate and reduce mtDNA loss during mitochondrial protein stress. a) Growth rates of *ρ^+^* (green dots) and *ρ*^0^ (red dots) cells bearing gene dosage increase plasmids. Dots represent independent biological replicates, means are represented as black bars. *P* values from a 1-tailed *t*-test comparing the *ρ*^0^ growth rates. b) Assay of mitochondrial membrane potential by TMRM fluorescence. Dots indicate population mean fluorescence from independent biological replicates consisting of at least 10,000 cells each, measured by flow cytometry. Fluorescence levels normalized to the *ρ^+^* control strain. Two-tailed *t*-test between the *ρ*^0^ control and each *ρ*^0^ group yielded *P* > 0.05 (not significant). c) Fluorescence micrograph of cells bearing gene dosage increase plasmids, expressing either mitoCherry or mitoFluc. d) Growth rates of cells bearing gene dosage increase plasmids in the presence and absence of mitochondrial protein stress. Significance figures are from a 2-tailed *t*-test between each group. e) mtDNA loss rate of cells bearing gene dosage increase plasmids during mitochondrial protein stress. Each dot represents petite fraction of an independent biological replicate. Black circles show population-weighted mean of 8 replicates for each genotype. Error bars represent simple, unweighted standard error between replicates. *P* values from 2-tailed *t*-tests between indicated genotypes.

Unexpectedly, several of the genes that were included in the above growth rate screen on the basis of a maladaptive ranking in the barcode sequencing screen grew substantially faster than the control in the growth rate screen, indicating a fitness gain. Six MoBY plasmids, *ECM22*, *FIG4*, *OSH3*, *YNL144C*, *VMA2*, and *SCY1*, showed this contradictory activity. Of these, all except *VMA2* were linked in literature databases to sterol or phosphoinositide-related biological processes (Supplementary Table 2). We selected the mtDNA stress dosage suppressors *ECM22*, *OSH3*, and *SCY1* from the putatively lipid-related group and *MIA40* and *PBP1* as representatives from the non-lipid group and profiled several phenotypes in order to characterize the cellular mechanisms of mtDNA stress resistance. We first regenerated *ρ*^0^ strains for each gene dose increase clone and again measured their growth rates to control for any confounding mutations that may have arisen during the growth rate screen. The growth rates of the *ρ*^0^ cells were consistent with the screening results and reproducibly grew faster than a control strain bearing a plasmid with no additional ORF (Fig. 5a). We hypothesized that dosage suppressors of mtDNA stress may act by boosting the membrane potential of *ρ*^0^ cells. To test this, we eliminated mtDNA from yeast cells bearing either a control plasmid or MoBY plasmid for each of the above genes by treatment with EtBr and incubated them with tetramethylrhodamine methyl ester (TMRM), which stains mitochondria in proportion to membrane potential. We then measured TMRM fluorescence by flow cytometry. Although mtDNA elimination produced a substantial depletion of TMRM staining, none of the dosage suppressors significantly altered TMRM fluorescence compared to the control (Fig. 5b).

Because the dosage suppressors enhanced growth, we wondered whether they reduced protein aggregation in mitochondria. We imaged mitoFluc and control cells bearing the dosage suppressors and assessed aggregate morphology (Fig. 5c). All strains still formed visible aggregates in the presence of the rescue alleles, demonstrating that they do not function by preventing protein aggregation or increasing aggregate clearance.

Considering that mitochondrial protein stress imparts a cellular growth defect by inducing mtDNA loss, we tested whether suppressors of mtDNA stress would reduce mitochondrial protein stress phenotypes. We transformed the aggregation-prone mitoFluc strain and control mitoCherry strain with the dosage increase plasmids identified previously and measured their growth rates. These suppressor constructs all increased the growth rates of cells expressing mitoFluc, though not to the level of unstressed control cells (Fig. 5d).

We next tested whether the mtDNA stress suppressors impacted the rate of mtDNA loss during protein stress in addition to their acceleration of cell growth after mtDNA elimination. We subjected control and mitoFluc cells bearing the suppressor plasmids to aerobic conditioning as described above to select for *ρ^+^* cells and then plated them on glucose medium (YPD) to relax the selection for respiration. We then replated these single colonies and counted the resulting petite fraction arising from each. Surprisingly, we found that all of the mtDNA stress suppressors reduced mtDNA loss during the outgrowth period, although the reduction in cells carrying the *MIA40* plasmid was not statistically significant (Fig. 5e). This plasmid was unique among those tested in that it raised the petite fraction in control cells that did not express mitoFluc. We conclude that suppressors of the growth defect resulting from mtDNA loss can also reduce mtDNA loss in *ρ^+^* cells during protein stress.

### SCY1 is a dosage-sensitive modulator of mtDNA homeostasis and is present in the mitochondrial matrix

**Fig. 6.**
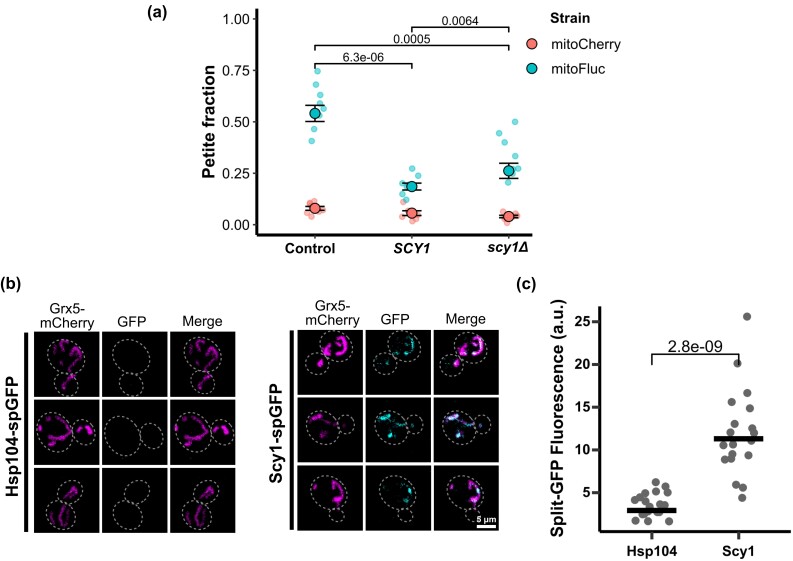
*SCY1* is a dosage-sensitive suppressor of mtDNA stress and is detected in the mitochondrial matrix. a) Petite frequency measurement by *SCY1* gene dosage. Dots are the petite fraction of independent biological replicates. Black circles show the weighted mean for each group of 8 replicates. Error bars indicate unweighted standard error between replicates. *P* values from 2-tailed *t*-test between only the mitoFluc groups. b) Representative fluorescence images of split-GFP localization assay. c) Fluorescence quantification of split-GFP assay for mitochondrial localization. Each dot represents the sum of split-GFP pixel intensities within a mask created by thresholding the mitochondrial matrix marker of 1 cell. Black bars show group mean. *P* value from 2-tailed *t*-test.

Of the strains we tested, *SCY1* gene dose increase was the most potent suppressor of mtDNA loss during mitochondrial protein stress. To further characterize the relationship between *SCY1* gene dose and susceptibility to mtDNA elimination, we knocked out the *SCY1* ORF to generate *scy1Δ* and again measured the mtDNA loss rate during mitochondrial unfolded protein stress relative to the wild type and cells bearing the additional allele on a plasmid. Unexpectedly, both increased *SCY1* gene dose and *scy1Δ* significantly suppressed mtDNA loss caused by mitoFluc expression (Fig. 6a).

Considering that mitoFluc aggregates and mtDNA both reside in the mitochondrial matrix, we wondered whether any Scy1 protein is imported into mitochondria. GFP-tagged Scy1 appears to localize to the late Golgi (Huh *et al*. 2003), but if it localizes to mitochondria at a concentration equal to or less than that of the cytoplasm, this fraction would not be apparent by microscopy. We therefore employed split-GFP labeling to detect only Scy1 protein that may be present in the mitochondrial matrix (Cabantous *et al*. 2005; Ruan *et al*. 2017). We first fused GFP β-strands 1–10 to the mitochondrial matrix protein Grx5. We then tagged Scy1 with GFP β-strand 11, such that any Scy1-GFP11 in the same cellular compartment as Grx5-GFP1-10 would complete the GFP structure and fluoresce. We used the same system to tag Hsp104p, an exclusively cytoplasmic protein, as a negative control. We found that in contrast to Hsp104-GFP11, Scy1-GFP11 produced a signal that was enriched in mitochondria, indicating localization to the mitochondrial matrix (Fig. 6, b and c). However, we do not believe that Scy1 is enriched in mitochondria, as previous studies also revealed the localization of Scy1 in cytoplasm and Golgi (Huh *et al*. 2003).

## Discussion

In this study, we showed that mitochondrial unfolded protein stress increases the rate of mtDNA loss in budding yeast and produces a population fraction that is unable to respire. We measured the mtDNA loss rate in stressed and non-stressed cells and determined the growth rates of yeast strains before and after elimination of mtDNA. This showed that mtDNA loss accounts for much of the growth defect observed in yeast cells during mitochondrial protein stress, and that the growth rate of cell populations bearing misfolded mitochondrial protein aggregates can be restored by enriching them in *ρ^+^* cells through selection on non-fermentable, respiration-obligate growth medium. Considering that *ρ^+^* cells under mitochondrial protein stress have a growth rate equal to non-stressed cells, that *ρ^+^* cells grow more quickly than *ρ*^0^ cells irrespective of protein stress, and that *ρ*^0^ cells under mitochondrial unfolded protein stress grow more slowly than non-stressed *ρ*^0^ cells, we propose that the burden of unfolded proteins within mitochondria is only detrimental insofar as it induces mtDNA loss and subsequently depresses the growth of *ρ*^0^ cells.

In addition to selecting for respiring cells, respiratory growth conditions are known to increase mtDNA copy number (Galeota-Sprung *et al*. 2022) and alter gene expression (Gasch *et al*. 2000). Thus, the fitness gain seen in the aerobically conditioned cells may also be due to residually amplified mtDNA or changes in gene expression or cellular structure that persist through more than 6 generations of recovery growth in glucose media that preceded the growth assays. Because loss or mutation of all of the mtDNA in a cell is irreversible, it can cause lasting changes in metabolism that do not respond to changes in nutrient availability.

Our 2-state growth model is consistent with several basic features of mtDNA stress and describes conditions under which *ρ*^0^ cells can reach a stable equilibrium in a population. However, there are several limitations to this scheme. The first is that this system describes a steady state, where stress induction is constant and growth conditions are fixed. Acute protein stresses, such as a heat shock that cells can recover from (as explored in Ruan *et al*. 2017) are expected to produce different effects. Similarly, the rates described here are all susceptible to changes in the growth medium. A culture that is not maintained in the exponential phase by nutrient repletion will exhibit selection for respiratory competence. Another limitation of the model is that we describe mtDNA presence or absence as a binary distinction, but copy number is likely relevant. Respiratory ability, among other phenotypes, may vary with the quantity of mtDNA that an individual cell contains. Future studies of mtDNA depletion in cell populations will benefit from single-cell DNA quantitation, which can determine the population distribution of mtDNA copy number and draw single-cell phenotype associations accordingly.

Mitochondrial gene expression is distinct in that DNA replication, transcription, and translation all occur in the same compartment. We found that several mtDNA-binding proteins and an ectopic nuclear DNA-binding domain all localized to mitochondrial unfolded protein aggregates, even when the aggregates were not colocalized with mtDNA nucleoids. This is consistent with our previous study, in which we subjected purified mitoFluc aggregates to proteomic mass spectrometry and found that they contained many native mitochondrial proteins (Ruan *et al*. 2020). Protein domains that interact with the hydrophobic nucleobase surface may be more susceptible to interaction with unfolded polypeptides, which have their lipophilic core exposed. Displacement of these proteins from mtDNA may perturb mtDNA replication, expression, or inheritance during cell division and give rise to *ρ*^0^ cells. Other studies have shown that impaired protein import destabilizes mtDNA (Weidberg and Amon 2018; Coyne and Chen 2019), and unfolded proteins in the mitochondrial matrix may similarly induce mtDNA loss by interrupting protein import.

The screen for dosage suppressors of *ρ*^0^ stress identified several adaptative genes reported in previous literature as well as several novel genes. Increased gene dosage of each of 2 histone core proteins was injurious to growth in the presence of EtBr. Yeast and most other species possess multiple copies of the histone genes, and previous studies have shown that sensitivity to DNA damage varies with histone gene dose, such that cells with additional histone copies are more susceptible to genotoxic agents (Liang *et al*. 2012). The gene dosage increases that we found to enhance *ρ*^0^ growth are involved in disparate cellular processes. Vts1 is an intrinsically disordered RNA-binding protein that forms the non-amyloid [SMAUG+] prion when overexpressed (Chakravarty *et al*. 2020). Yck1 and Yck3 are homologous casein kinases and are involved in glucose sensing and repression of respiratory metabolism. Their paralog *YCK2* was also enriched in the screen, but to a lesser degree (Supplementary Table 1). It is curious that gene dose increases from the lipid-related cluster were maladaptive in the barcode sequencing screen yet some of the most potent adaptations in the growth rate screen. This discrepancy may be related to the fact that the barcode sequencing screen was conducted in large, well-shaken flasks rather than small, sealed assay plates. It may also be relevant that the cells in the barcode sequencing experiment were allowed to reach the diauxic shift and approach the stationary phase before passage, whereas growth rates were measured strictly within the exponential growth phase. Nevertheless, these lipid-related genes were confirmed to be potent suppressors of *ρ*^0^ stress in subsequent growth assays. These gene products may modulate the lipid composition of the mitochondrial inner membrane to promote mtDNA stability. Yeast cells accumulate cardiolipin during chronic heat stress (Luévano-Martínez *et al*. 2015), yet mutants that are deficient in phosphatidylethanolamine and cardiolipin biosynthesis are prone to mtDNA loss (Birner *et al*. 2001; Chen *et al*. 2010) and are sensitized to mtDNA depletion during heat shock.

Interestingly, gene dose increases that aided cellular growth following mtDNA loss also prevented mtDNA loss during mitochondrial protein stress. This may be explained by the fact that mtDNA homeostasis is interdependent with many mitochondrial functions, and disrupting any of them can cause cascading failures. For example, respiration and membrane potential maintenance depend on mtDNA replication, which itself relies on the membrane potential to import the components required for DNA synthesis. Because these dosage effects are adaptive in *ρ*^0^ cells, which are severely deficient in many normal mitochondrial functions, it appears that they can compensate for mitochondrial functions without also depending on functional mitochondria. Breaking this loop of interdependence is expected to increase the robustness of the system to perturbations such as protein misfolding. These are, to our knowledge, the first known suppressors of mtDNA loss. Subsequent investigations should identify how these genes act to spare mtDNA from mitochondrial protein stress and determine whether they improve resistance to other stressors, especially in disease models related to protein homeostasis, aging, hyperglycemia, and ischemic injury, where mtDNA depletion is observed.

Increasing *SCY1* gene dosage reduced mtDNA loss during mitochondrial protein stress to a greater degree than the other mutations that we tested. Surprisingly, *SCY1* knockout also protected against mtDNA loss, demonstrating that *SCY1* gene dose is not associated with mitochondrial homeostasis in a simple, linear way. Synonymous point mutations in the human homolog *SCY1*-like 1 (*SCYL1*) cause an mRNA splicing defect associated with recessive familial cerebellar ataxia (Shohet *et al*. 2019), providing further evidence that subtle changes to the expression mechanism of *SCY1*-like genes can produce striking phenotypes. Inactivation of *SCYL1* characterizes the muscle-deficient (*mdf*) mouse line, which serves as a model of progressive motor neuropathy (Burman *et al*. 2008). However, the mechanism by which *SCYL1* dysfunction contributes to neurodegeneration remains elusive. Like yeast *SCY1*, its principal domain is a pseudokinase that lacks conserved residues that are necessary for phosphotransferase activity (Pelletier 2016). Its role in mtDNA maintenance is also unclear. One study found that injecting mtDNA-intact mitochondria into the cytoplasm of mtDNA-depleted pig oocytes altered *SCYL1* expression (Tsai *et al*. 2018), while other research implicates the gene in COPI-coated vesicle trafficking (Burman *et al*. 2008). Our finding that Scy1 protein is detectable in the mitochondrial matrix raises the question whether it serves some function there. We also wonder whether neurodegeneration in *SCYL1*-disrupted animals (Schmidt *et al*. 2007) is mediated by depletion of functional mtDNA. Future studies should explore how variations in *SCY1* and *SCYL1* gene dosage, knockout, and even conservative point mutations give rise to markedly different phenotypes, and examine the connection between these variants and mtDNA maintenance.

## Supplementary Material

jkad272_Supplementary_Data

## Data Availability

Raw sequencing data have been deposited at the NCBI Sequence Read Archive (https://www.ncbi.nlm.nih.gov/sra) with the accession number PRJNA945832, BioSample SAMN33794339. Data analysis scripts are available at: https://github.com/RongLiLab/McNamara-et-al-2023. Supplemental material available at G3 online.
