# Supplementary material for: Gene dosage adaptations to mtDNA depletion and mitochondrial protein stress in budding yeast
Source: G3 (Bethesda). 2023 Dec 21;14(2):jkad272. doi: 10.1093/g3journal/jkad272 (PMC10849340; doi:10.1093/g3journal/jkad272)
Supplement: jkad272_Supplementary_Data [file jkad272_supplementary_data.zip › Figure_S2_G3-2023-404544.pdf]

Variables

$\mathrm{P_{wt}}$ = Population proportion of wild-type cells

$\mathrm{P_{mut}}$ = Population proportion of mutant cells

$\mathrm{G_{wt}}$ = Exponential growth rate of wild-type cells

$\mathrm{G_{mut}}$ = Exponential growth rate of mutant cells

Differential equations for population proportion

$\dot{P}_{\mathrm{wt}} = G_{\mathrm{wt}} P_{\mathrm{wt}} - r P_{\mathrm{wt}}$

$\dot{P}_{\mathrm{mut}} = G_{\mathrm{wt}} P_{\mathrm{mut}} + r P_{\mathrm{wt}}$

Linear systems rewritten as:

$\dot{\vec{P}} = A \cdot \vec{P}$

$\vec{P} = (P_{\mathrm{wt}}, P_{\mathrm{mut}})$

$$\mathrm{A}=\begin{pmatrix} G_{\mathrm{wt}} - r & 0 \\ r & G_{\mathrm{mut}} \end{pmatrix}$$

Solve by finding eigenvectors and eigenvalues of matrix A:

$\vec{v}_1 = \left(0, \frac{r}{G_{\mathrm{wt}} - G_{\mathrm{mut}} - r}\right)$ for eigenvalue $\lambda_1 = G_{\mathrm{mut}}$

$\vec{v}_2 = \left(1, \frac{r}{G_{\mathrm{wt}} - G_{\mathrm{mut}} - r}\right)$ for eigenvalue $\lambda_2 = G_{\mathrm{wt}} - r$

Yielding:

$\vec{P} = c_1 e^{\lambda_1 t} \vec{v}_1 + c_2 e^{\lambda_2 t} \vec{v}_2$

Assuming a starting population of 1 for wild-type and 0 for the mutant, we assign constants

$\mathrm{c_1} = -1$

$c_2 = 1$

Obtaining expressions for population proportion as a function of time:

$P_{\mathrm{wt}} = e^{(G_{\mathrm{wt}}-r)t}$

$P_{\mathrm{mut}} = \frac{r}{G_{\mathrm{wt}}-G_{\mathrm{mut}}-r}\left(e^{(G_{\mathrm{wt}}-r)t} - e^{G_{\mathrm{mut}}t}\right)$
