# Supplementary material for: Gene dosage adaptations to mtDNA depletion and mitochondrial protein stress in budding yeast
Source: G3 (Bethesda). 2023 Dec 21;14(2):jkad272. doi: 10.1093/g3journal/jkad272 (PMC10849340; doi:10.1093/g3journal/jkad272)
Supplement: jkad272_Supplementary_Data [file jkad272_supplementary_data.zip › Supplemental_Material_Legends_G3-2023-404544.docx]

**Figure S1.** DAPI staining of cells from respiring and non-respiring colonies. Respiring colonies show cytoplasmic DAPI foci, indicative of mtDNA.

**Figure S2.** Derivation of the two-state growth model of mtDNA loss in a cell population.

**Table S1.** Key to gene barcode sequences, key to experiment ID barcode sequences, read counts for each indexed barcode. Differential enrichment score for each plasmid following EtBr treatment and following *ρ^0^* growth.

**Table S2.** Screen results for lipid-related gene cluster and gene descriptions from SGD (<https://www.yeastgenome.org/>).

**Table S3.** Yeast strains used in this study.

**Table S4.** Plasmids used in this study.

**Table S5.** Primers used for sequencing library preparation.
