# Supplementary material for: Gene dosage adaptations to mtDNA depletion and mitochondrial protein stress in budding yeast
Source: G3 (Bethesda). 2023 Dec 21;14(2):jkad272. doi: 10.1093/g3journal/jkad272 (PMC10849340; doi:10.1093/g3journal/jkad272)
Supplement: jkad272_Supplementary_Data [file jkad272_supplementary_data.zip › Table_S2_G3-2023-404544.pdf]

Gene	Common name	Rank metric	rho0 growth rate (hr^-	rho+ growth rate (hr^-1)	Description
YHR073W	OSH3	-85	0.50	0.67	Member of an oxysterol-binding protein family; this family has seven members in S. cerevisiae; family members have overlapping, redundant functions in sterol metabolism and collectively perform a function essential for viability; contains FFAT motif; interacts with ER anchor Scs2p at patches at the plasma membrane; regulated by sterol binding
YNL325C	FIG4	-58	0.48	0.70	Phosphatidylinositol 3,5-bisphosphate (PtdIns[3,5]P) phosphatase; required for efficient mating and response to osmotic shock; physically associates with and regulated by Vac14p; contains a SAC1-like domain; homologous to human FIG4, which is associated with CMT4J, a form of Charcot-Marie-Tooth disorder
YLR228C	ECM22	-60	0.47	0.71	Sterol regulatory element binding protein; regulates transcription of sterol biosynthetic genes upon sterol depletion, after relocating from intracellular membranes to perinuclear foci; redundant activator of filamentation with UPC2, up-regulating the expression of genes involved in filamentous growth; contains Zn[2]-Cys[6] binuclear cluster; ECM22 has a paralog, UPC2, that arose from the whole genome duplication
YGL083W	SCY1	-58	0.47	0.67	Putative kinase; suppressor of GTPase mutant; similar to bovine rhodopsin kinase; may have a role in intracellular sterol transport
YNL144C	YNL144C	-55	0.46	0.68	Putative protein of unknown function; non-tagged protein is detected in highly purified mitochondria in high-throughput studies; contains a PH domain and binds phosphatidylinositols and phosphatidylethanolamine in a large-scale study; YNL144C has a paralog, YHR131C, that arose from the whole genome duplication
Control plasmid	–	-	0.45	0.62	-
