# Supplementary material for: Gene dosage adaptations to mtDNA depletion and mitochondrial protein stress in budding yeast
Source: G3 (Bethesda). 2023 Dec 21;14(2):jkad272. doi: 10.1093/g3journal/jkad272 (PMC10849340; doi:10.1093/g3journal/jkad272)
Supplement: jkad272_Supplementary_Data [file jkad272_supplementary_data.zip › Table_S3_G3-2023-404544.docx]

| **ID** | **Alias** | **Background** | **Genotype** | **Source** |
| --- | --- | --- | --- | --- |
| RLY10200 | WT | BY4741 | BY4741, WT | Rong Li Lab stock |
| RLY10201 | mitoCherry | BY4741 | BY4741, trp::Su9-mCherry::NatMx | Ruan et al. 2020 |
| RLY10202 | mitoFluc | BY4741 | BY4741, trp::Su9-FlucSM-mCherry::NatMx | Ruan et al. 2020 |
| RLY10203 | Tom70-GFP mitoFluc | BY4741 | BY4741, Tom70-GFP::HisMx, trp::Su9-FlucSM-mCherry::NatMx | Ruan et al. 2020 |
| RLY10204 | mtDNA_LacO, LacI-3xGFP | W303 | W303 (ATCC201238), Mat a/alpha, leu2-3,112/leu2-3,112 trp1-1/TRP1 can1-100/ can1-100 ura3-1 ade2-1/ADE2 his3-11,15 HO-PCup-mt-3xGFP-LacI-HO, pvt100u-mt-dsRed, [mtDNA_LacO::COX2] | Peter Walter Lab, PWY1933 |
| RLY10205 | kar1-1 MATalpha | see Conde & Fink, 1976 | MATalpha, his4-15, kar1-1 | Conde & Fink 1976, via Reed Wickner #1020 |
| RLY10206 | W303 mtDNA_LacO, LacI-3xGFP | W303 | W303 (ATCC201238), Mat-a, leu2-3,112/leu2-3,112 trp1-1/TRP1 can1-100/ can1-100, ura3-1 ade2-1/ADE2 his3-11,15 HO-PCup-mt-3xGFP-LacI-HO, pvt100u-mt-dsRed, [mtDNA_LacO::COX2] | Sporulated from RLY10204 |
| RLY10207 | BY LacO | BY4741 | BY4741, mat-a, HO::PCup-mt-3xGFP-LacI::KanMX::HO, [mtDNA_LacO::COX2] | This study |
| RLY10208 | BY LacO mitoCherry | BY4741 | BY4741, mat-a, HO::PCup-mt-3xGFP-LacI::KanMX::HO, trp::Su9-mCherry::NatMx, [mtDNA_LacO::COX2] | This study |
| RLY10209 | BY LacO mitoFluc | BY4741 | BY4741, mat-a, HO::PCup-mt-3xGFP-LacI::KanMX::HO, trp::Su9-FlucSM-mCherry::NatMx, [mtDNA_LacO::COX2] | This study |
| RLY10210 | mitoCherry Rim1-GFP | BY4741 | BY4741, Rim1-GFP::HisMx, trp::Su9-mCherry::NatMx | This study |
| RLY10211 | mitoCherry Kgd2-GFP | BY4741 | BY4741, Kgd2-GFP::HisMx, trp::Su9-mCherry::NatMx | This study |
| RLY10212 | mitoCherry Pim1-GFP | BY4741 | BY4741, Pim1-GFP::HisMx, trp::Su9-mCherry::NatMx | This study |
| RLY10213 | mitoFluc Rim1-GFP | BY4741 | BY4741, Rim1-GFP::HisMx, trp::Su9-FlucSM-mCherry::NatMx | This study |
| RLY10214 | mitoFluc Kgd2-GFP | BY4741 | BY4741, Kgd2-GFP::HisMx, trp::Su9-FlucSM-mCherry::NatMx | This study |
| RLY10215 | mitoFluc Pim1-GFP | BY4741 | BY4741, Pim1-GFP::HisMx, trp::Su9-FlucSM-mCherry::NatMx | This study |
| RLY10216 | Scy1-KO mitoCherry | BY4741 | BY4741 ∆scy1::KanMX, trp::Su9-mCherry::NatMx | This study |
| RLY10217 | Scy1-KO mitoFluc | BY4741 | BY4741 ∆scy1::KanMX, trp::Su9-FlucSM-mCherryNatMx | This study |
| RLY10218 | Hsp104-spGFP | BY4741 | BY4741 trp1::GPD-Grx5-GFP1-10::natMX6, Tom70-mCherry::URA3, Hsp104-GFP11::HisMx | This study |
| RLY10219 | Scy1-spGFP | BY4741 | BY4741 trp1::GPD-Grx5-GFP1-10::natMX6, Tom70-mCherry::URA3, Scy1-GFP11::HisMx | This study |
