# Supplementary material for: Gene dosage adaptations to mtDNA depletion and mitochondrial protein stress in budding yeast
Source: G3 (Bethesda). 2023 Dec 21;14(2):jkad272. doi: 10.1093/g3journal/jkad272 (PMC10849340; doi:10.1093/g3journal/jkad272)
Supplement: jkad272_Supplementary_Data [file jkad272_supplementary_data.zip › Table_S4_G3-2023-404544.docx]

| **ID** | **Description** | **Use** |
| --- | --- | --- |
| RLB119 | pFA6a_KanMX2 | Gene knockout |
| RLB451 | pFA6a_GFP_HIS | GFP tagging |
| RLB917 | pFA6a_GFP11_HIS | Split-GFP tagging |
| RLB971 | Trp_Su9-FlucSM-mCherry_NatMx | Mitochondrial protein stress |
| RLB973 | Trp_Su9-mCherry_NatMx | Mitochondrial matrix marker |
| RLB1105 | HO_LacI-3xGFP_KanMx_HO | mtDNA imaging |
| RLB1106 | MoBY empty control plasmid | Control |
| See Ho et al. 2009 | MoBY Library | Various |
