# Supplementary material for: Gene dosage adaptations to mtDNA depletion and mitochondrial protein stress in budding yeast
Source: G3 (Bethesda). 2023 Dec 21;14(2):jkad272. doi: 10.1093/g3journal/jkad272 (PMC10849340; doi:10.1093/g3journal/jkad272)
Supplement: jkad272_Supplementary_Data [file jkad272_supplementary_data.zip › Table_S5_G3-2023-404544.docx]

| **Primer ID** | **Description** | **Illumina adapter sequence** | **Experiment ID** | **Barcode UPTAG primer** | **Primer sequence** |
| --- | --- | --- | --- | --- | --- |
| josh_ilmn_001 | F primer | CAAGCAGAAGACGGCATACGAGAT | cgctcagttc | GATGTCCACGAGGTCTCT | CAAGCAGAAGACGGCATACGAGATcgctcagttcGATGTCCACGAGGTCTCT |
| josh_ilmn_002 | F primer | CAAGCAGAAGACGGCATACGAGAT | tatctg | GATGTCCACGAGGTCTCT | CAAGCAGAAGACGGCATACGAGATtatctgGATGTCCACGAGGTCTCT |
| josh_ilmn_003 | F primer | CAAGCAGAAGACGGCATACGAGAT | atatga | GATGTCCACGAGGTCTCT | CAAGCAGAAGACGGCATACGAGATatatgaGATGTCCACGAGGTCTCT |
| josh_ilmn_004 | F primer | CAAGCAGAAGACGGCATACGAGAT | cttatg | GATGTCCACGAGGTCTCT | CAAGCAGAAGACGGCATACGAGATcttatgGATGTCCACGAGGTCTCT |
| josh_ilmn_005 | F primer | CAAGCAGAAGACGGCATACGAGAT | taatct | GATGTCCACGAGGTCTCT | CAAGCAGAAGACGGCATACGAGATtaatctGATGTCCACGAGGTCTCT |
| josh_ilmn_006 | F primer | CAAGCAGAAGACGGCATACGAGAT | gcgcga | GATGTCCACGAGGTCTCT | CAAGCAGAAGACGGCATACGAGATgcgcgaGATGTCCACGAGGTCTCT |
| josh_ilmn_007 | F primer | CAAGCAGAAGACGGCATACGAGAT | agagca | GATGTCCACGAGGTCTCT | CAAGCAGAAGACGGCATACGAGATagagcaGATGTCCACGAGGTCTCT |
| josh_ilmn_008 | F primer | CAAGCAGAAGACGGCATACGAGAT | tgcctt | GATGTCCACGAGGTCTCT | CAAGCAGAAGACGGCATACGAGATtgccttGATGTCCACGAGGTCTCT |
| josh_ilmn_009 | F primer | CAAGCAGAAGACGGCATACGAGAT | ctactc | GATGTCCACGAGGTCTCT | CAAGCAGAAGACGGCATACGAGATctactcGATGTCCACGAGGTCTCT |
| josh_ilmn_010 | F primer | CAAGCAGAAGACGGCATACGAGAT | tcgtct | GATGTCCACGAGGTCTCT | CAAGCAGAAGACGGCATACGAGATtcgtctGATGTCCACGAGGTCTCT |
| josh_ilmn_011 | F primer | CAAGCAGAAGACGGCATACGAGAT | gaacat | GATGTCCACGAGGTCTCT | CAAGCAGAAGACGGCATACGAGATgaacatGATGTCCACGAGGTCTCT |
| josh_ilmn_012 | F primer | CAAGCAGAAGACGGCATACGAGAT | cctatg | GATGTCCACGAGGTCTCT | CAAGCAGAAGACGGCATACGAGATcctatgGATGTCCACGAGGTCTCT |
| josh_ilmn_013 | F primer | CAAGCAGAAGACGGCATACGAGAT | taatgg | GATGTCCACGAGGTCTCT | CAAGCAGAAGACGGCATACGAGATtaatggGATGTCCACGAGGTCTCT |
| josh_ilmn_014 | F primer | CAAGCAGAAGACGGCATACGAGAT | gtgccg | GATGTCCACGAGGTCTCT | CAAGCAGAAGACGGCATACGAGATgtgccgGATGTCCACGAGGTCTCT |
| josh_ilmn_015 | F primer | CAAGCAGAAGACGGCATACGAGAT | cggcaa | GATGTCCACGAGGTCTCT | CAAGCAGAAGACGGCATACGAGATcggcaaGATGTCCACGAGGTCTCT |
| josh_ilmn_016 | F primer | CAAGCAGAAGACGGCATACGAGAT | gccgta | GATGTCCACGAGGTCTCT | CAAGCAGAAGACGGCATACGAGATgccgtaGATGTCCACGAGGTCTCT |
| josh_ilmn_017 | F primer | CAAGCAGAAGACGGCATACGAGAT | aaccat | GATGTCCACGAGGTCTCT | CAAGCAGAAGACGGCATACGAGATaaccatGATGTCCACGAGGTCTCT |
| josh_ilmn_018 | F primer | CAAGCAGAAGACGGCATACGAGAT | ggttgc | GATGTCCACGAGGTCTCT | CAAGCAGAAGACGGCATACGAGATggttgcGATGTCCACGAGGTCTCT |
| josh_ilmn_R02 | R primer Illumina Read 1 seq | AATGATACGGCGACCACCGAGATCT | - | GTCGACCTGCAGCGTACG | AATGATACGGCGACCACCGAGATCTGTGACACTCTTTCCCTACACGACGCTCTTCCGATCTGTCGACCTGCAGCGTACG |
