# Supplementary figures and images for: Gene dosage adaptations to mtDNA depletion and mitochondrial protein stress in budding yeast
Source: G3 (Bethesda). 2023 Dec 21;14(2):jkad272. doi: 10.1093/g3journal/jkad272 (PMC10849340; doi:10.1093/g3journal/jkad272)

## Respiratory growth

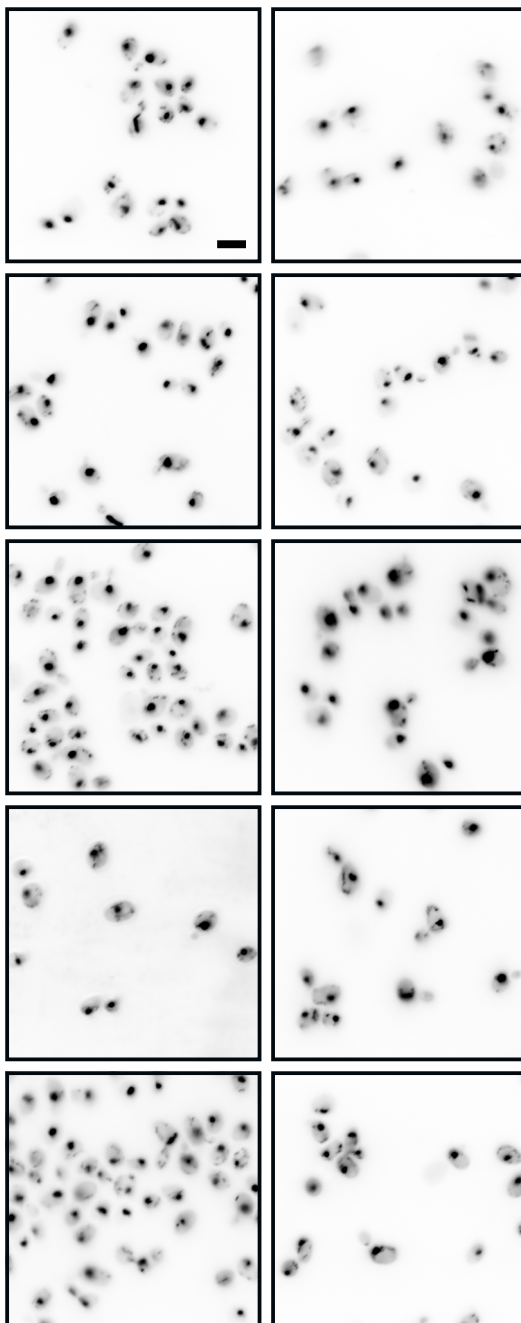

## No respiratory growth

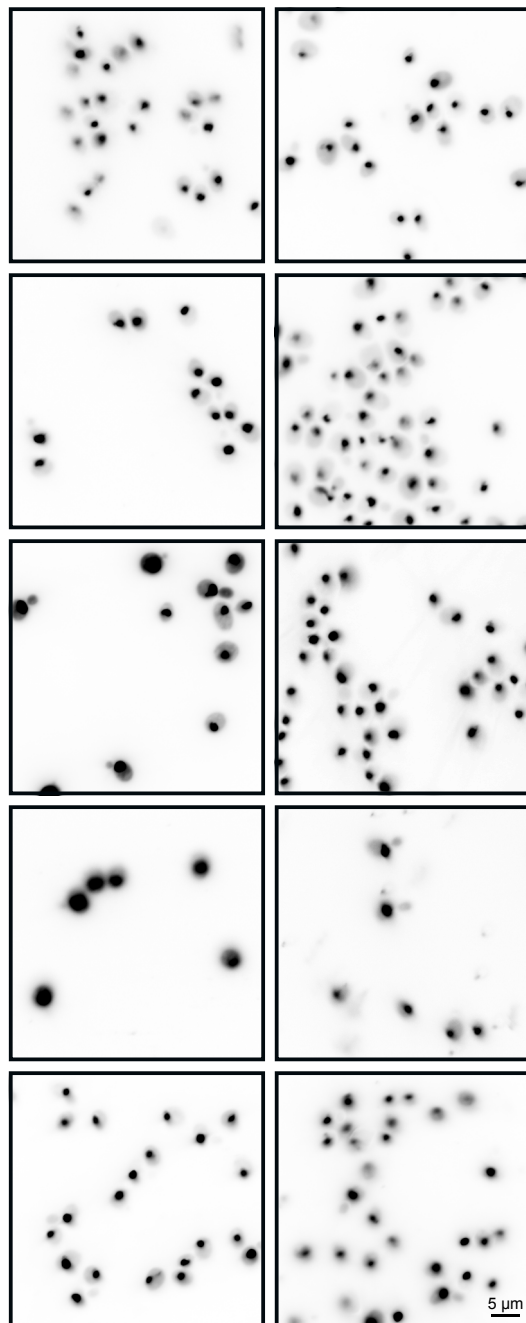

Supplement: jkad272_Supplementary_Data [file jkad272_supplementary_data.zip › Figure_S1_G3-2023-404544.pdf]
